# Patient safety culture in primary health care: Medical office survey on patient safety culture in a Brazilian family health strategy setting

**DOI:** 10.1371/journal.pone.0271158

**Published:** 2022-07-26

**Authors:** Gleiton Lima Araújo, Fábio Ferreira Amorim, Rafaela Cristina Pereira Santos de Miranda, Flávio Ferreira Pontes Amorim, Levy Aniceto Santana, Leila Bernarda Donato Göttems

**Affiliations:** 1 Postgraduation Program in Health Sciences, School of Health Sciences (ESCS), Brasília, Federal District, Brazil; 2 School of Health Sciences (ESCS), Brasília, Federal District, Brazil; 3 Universidade Católica de Brasília (UCB), Brasília, Federal District, Brazil; Universita degli Studi di Milano, ITALY

## Abstract

**Study objectives:**

To assess the patient safety culture in Primary Health Care (PHC) setting after the transition to the Family Health Strategy (FHS) model in a Brazilian metropolitan area and compare the results between the categories of health care professionals.

**Methods:**

A cross-sectional study including 246 workers from primary health care services in Federal District, Brazil. Data collection took place from October to December 2019 through the Medical Office Survey on Patient Safety Culture (MOSPSC) application. Patient safety culture was considered positive when the score was above 60%. For comparisons between the categories of health care professionals’, the ANOVA and Kruskal-Wallis test were used for composite percent positive scores, and Pearson’s chi-square or Fishers exact test for frequency and percentage of positive responses.

**Results:**

The overall MOSPSC composite percent positive score was 49.9%. Among the 12 dimensions, only three showed a positive patient safety culture: Teamwork (73.1%), Organizational learning (62.9%), and Patient care tracking/follow-up (60.1%). The percentage of positive responses on overall quality assessment (78.1%) and overall patient safety assessment (78.0%) showed a positive evaluation. There was no significant difference in the composite percent positive score of overall MOSPSC (p = 0.135) and the percentage of positive responses on overall patient safety assessment (p = 0.156) between the categories of health care professionals. Overall quality assessment showed a significant difference between job roles (p < 0.001), in which nursing /health care technicians showed a significantly lower score than other job roles.

**Conclusion:**

The patient safety culture assessment showed a weakness in the patient safety in the PHC services. The MOSPSC and nine of its dimensions presented a negative safety culture assessment, regardless of the high scores in the overall patient safety and quality assessments.

## Introduction

Patient safety can be defined as preventing evitable harm during health care delivery. When the World Health Organization (WHO) launched the World Alliance for Patient Safety in 2004, the countries have carried out many actions. This includes the development of global norms and standards and the promotion of evidence-based policies to increase patient safety worldwide [1, 2]. The health care organizations should promote a patient safety culture, a multidimensional concept and a product of individual and group values, attitudes, skills, and behavioral patterns, to determine the commitment, style, and proficiency of the health care service to protect patients from errors and adverse events [3–5]. Furthermore, interdisciplinary cooperation helps healthcare providers comprehend typical roles and duties, allowing them to execute organizational goals, communicate and distribute essential information, and offer safe and effective care [6]. According to the Organization for Economic Co-operation and Development (OECD), it is necessary to make the security flaws in Primary Health Care (PHC) visible, and managers and professionals must be committed to this concern. Besides, collaboration and cooperation, patient involvement in their care, the measurement and reporting of harm, and other outcomes are essential to implement and maintain a safety culture in PHC [7]. Indeed, health care organizations with a positive patient safety culture are characterized by communications based on mutual trust, shared perceptions of the importance of patient safety, and confidence in the effectiveness of the preventive measure [3, 4, 8].

Nowadays, patient safety culture has been widely discussed worldwide, a significant concern in health care services [9, 10]. PHC is a cornerstone and inseparable part of the health care systems, responsible for most health care delivery for patients over time [11, 12]. Although less emphasized, incidents related to health care delivery may also occur in PHC setting, and the commitment of managers and professionals regarding this issue is essential [5, 13]. In a previous systematic review, the main types identified of PHC incidents were administrative and communication, failures and delays in diagnostics, and mistakes in the administration of prescription and medication. The authors suggest that safety incidents are common, and most do not result in serious harm that reaches the patient [14]. However, diagnostic and prescription errors may be significant sources of iatrogenic damages, worsening the patients’ illnesses resulting in more complex health care resources that may require hospitalization [7]. Therefore, patient safety culture assessment is essential to prevent damage and improve patient care. It is the starting point for tracing actions and changes to reduce incidents providing a better health care quality [3, 5, 13]. In addition, these assessments should be used to monitor the patient safety culture in the health care services and the impact of patient safety programs over time [5, 13], improving patient safety in PHC settings by a constructive safety culture with agreed values, attitudes, and safe behaviors in everyday practice. It is critical to have a conversation about the topic within the team and in the context of institutional realities, involving all care providers professionals to be aware of their role in incident prevention during the care [15–18].

Although the health care in PHC setting differs from the hospital setting by the characteristics of the patients, the organizational structure, the relationships between health care professionals and patients, and the type and results of patient safety incidents, the majority of tools and instruments for assessment of safety culture has been mainly based on tools and instruments for evaluation of hospital care [3, 10, 19]. This consideration is an important issue as the risks associated with hospital-based care are not the same as those related to primary care [3, 18, 19]. The beliefs and practices regarding patient safety shared by the members of an organization determine its patient safety culture [15, 16]. The assessment of patient safety culture on the PHC services should consider the behaviors of health care professionals among their different job roles, including the unique features of PHC setting that is substantially different compared to the hospital setting [19].

Notwithstanding concerns about patient safety in primary health care have been raised only recently, among the patient safety culture assessments used in the PHC services, the Medical Office Survey on Patient Safety Culture (MOSPSC) was developed by the Agency for Healthcare Research and Quality (AHRQ) in the United States to be used in the extra-hospital setting as an extension of the hospital survey on patient safety culture [15, 20]. Although the MOSPSC may lead to lower sensitivity in the patient safety culture attitudinal dimensions than other surveys, such as the Safety Attitudes Questionnaire (SAQ), the MOSPSC also evaluates communication, managers’ perception, and teamwork. According to the World Health Organization, these are critical components of the positive safety culture [15, 20, 21].

Brazil has the most extensive free-of-charge public health system in the world, with nearly 160 million people (76% of the Brazilian population) using health care services of the Brazilian Unified Health Care System (SUS) [22]. In recent years, the Brazilian PHC was reorganized to replace most of PHC traditional units based on physician-centered care with general practitioners or specialists into the Family Health Strategy (FHS) model, emphasizing health care in community health services and at home. In the FHS model, family health teams provide services, including one physician, one nurse, one nursing assistant/technician, and four to 12 full-time community health workers (frontline public health workers). Each team is assigned with 2,000 to 3,500 people located within its territory, guaranteeing the principles and guidelines of PHC. The family health teams also may be supported by a dental team. In addition, each group of four or five family health teams also has other health care professionals such as psychologists, community pharmacists, and physiotherapists to provide additional specialist health care and support [22, 23].

The FHS made it possible to expand the PHC coverage to a large portion of the Brazilian inhabitants, especially for the most vulnerable, remote, and deprived populations, becoming a successful model for the public health care system improvement in the attempt to achieve the universal right to health [22, 24–27]. Moreover, considering the attributes of the FHS, such as comprehensiveness, longitudinally, family orientation, community orientation, and coordination, the FHS may be associated with a positive patient safety culture [27]. It should be highlighted that the community health workers are cornerstones of the FHS model since they bring and monitor the health care population because they live in the community where they work [23, 28].

Considering the Federal District, Brazil, replaced the traditional PHC units into FHS in 2017 [29], this study aimed to assess the patient safety culture using the MOSPSC in the PHC setting after changing to the FHS model. Furthermore, it seeks to reveal whether significant positive aspects are already observed, making it possible to produce relevant information in management to develop programs and actions to improve the patient safety culture in PHC services.

## Materials and methods

### Study setting and design

A cross-sectional study carried out from October to December 2019, using the MOSPSC, applied to health care professionals in the PHC services of the North health region of the Federal District, Brazil. The North health region, one of the nine health regions of the Federal District metropolitan area, serves nearly 380,000 inhabitants by its public health system, including 15 PHC units and 806 health care professionals.

### Participants

The study included a convenience sample of 246 health care professionals who were willing to participate when researchers visited PHC services to apply the survey.

### Procedures

The MOSPSC was a questionnaire developed in 2007 by the AHRQ to assess the patient safety culture in extra-hospital settings [15, 20]. The MOSPSC was translated to Brazilian Portuguese with cross-cultural adaptation and validated to evaluate the patient safety culture in the PHC setting, with Cronbach’s alpha = 0.95, expressing high reliability [30]. The authors that performed the translation, cross-cultural adaptation, and validation of the Brazilian Portuguese MOSPSC version authorized to use the survey in our study. S1 File shows the English and Portuguese MOSPSC versions.

The Brazilian Portuguese MOSPSC version consists of 52 items evaluated on a five or six-point Likert scale that measure 12 dimensions of the patient safety construct, including 1) Communication openness; 2) Communication about error; 3) Information exchange with other institutions; 4) Office processes and standardization; 5) Organizational learning; 6) Overall perceptions of patient safety and quality; 7) Overall ratings on quality and patient safety; 8) Owner/managing partner/leadership support for patient safety; 9) Patient care tracking/follow-up; 10) Staff training; 11) Teamwork; and 12) Work pressure and pace. Another five items (Patient-centered, Effective, Punctual, Efficient, and Impartial) on a five-point Likert scale performed the Overall quality assessment, and one item on a five-point Likert scale evaluated the Overall patient safety assessment. All items also included the response option Does not apply, or I don’t know [30]. Finally, three questions evaluate professional practices (job role, hours per week worked, and years worked in the PHC service).

### Data analysis

The MOSPSC analysis was performed according to the AHRQ recommendations. Responses with the highest scores on the Likert scale indicate a more positive patient safety culture evaluation at each item level. The codes of negatively worded items (identified in S1 File) were reversed before the responses were scored, and Does not apply, or I don’t know responses were treated as missing data (non-response). A positive response was considered when the response score was equal to or above four on any five-point or six-point Likert scale [22].

To data analysis of the 12 dimensions of the patient safety construct, a percent positive score at each item level was calculated (percentage of positive responses of the total of valid responses received in each item). Then, a percent positive score of each dimension was estimated using the mean percentage of positive responses of the items performed to evaluate the dimension. Finally, the composite percent positive score of the overall MOSPSC was calculated using the mean percent score of positive responses of all dimensions. Positive patient safety culture was considered when the composite percent positive score was above 60% for dimensions and overall MOSPSC. The dimensions were also classified as strength when they present a composite percent positive score equal to or greater than 75%, or weak point when the composite percent positive score was less than 60%, suggesting the need for improvements in the analyzed dimension [20].

The frequency and percentage of positive responses of the total of valid responses received in each assessment were computed to analyze the Overall quality assessment and the Overall patient safety assessment. The frequency and percentage of positive responses of the total of valid responses received were also evaluated for each item of the Overall quality assessment (Patient-centered, Effective, Punctual, Efficient, and Impartial). Positive assessment was considered when the percentage of positive responses was above 60% [20].

For statistical analysis, composite percent positive scores are expressed as mean ± standard deviation (SD) and the median and interquartile range (25–75%). According to the Kolmogorov–Smirnov test with Lilliefors correction, the overall MOSPSC composite percent positive score had a normal distribution, and all composite percent positive scores of MOSPSC dimensions had a non-normal distribution. Thus, analysis of variance (ANOVA) was used to compare overall MOSPSC composite percent positive scores between job roles. In addition, the Kruskal-Wallis test was used to compare composite percent positive scores of MOSPSC dimensions between job roles. Finally, when necessary, the post hoc analysis was performed using the Student’s t-test, or Mann-Whitney test, with Bonferroni correction as appropriate.

To compare the frequency and percentage positive responses on Overall patient safety assessment and Overall quality assessment and the items performed to evaluate the Overall quality assessment, contingency tables and Pearson’s chi-square test (χ2) or Fisher’s exact test were used as appropriate. In addition, when necessary, the post hoc analysis was performed using the chi-square goodness-of-fit test with Bonferroni correction.

Statistical analyses were performed using IBM Statistical Package for Social Sciences 23.0 for Mac (SPSS 23.0 Mac, SPSS Inc., Chicago, USA). The level of statistical significance was defined as a two-sided p-value ≤ 0.05.

### Ethics statement

The study was approved by the Research Ethics Committee of the Foundation of Teaching and Research in Health Sciences (FEPECS), Brasília, Distrito Federal, Brazil. All participants gave informed consent before completing the survey.

## Results

Out of 246 participants, 71 were nursing technicians/health care technicians (28.9%), 56 administrative staff (22.8%), 54 nurses (22.0%), 27 community health workers (11.0%), 22 physicians (8.9), and 16 dentists (6.5%). Two hundred twenty-eight worked 20–40 hours weekly in the PHC service (92.7%), and 138 worked in the PHC service for six years or more (Table 1).

**Table 1:** Health care professionals, according to the job role, hours worked per week and years worked in the primary health care service (n = 246).

Job role, n (%)	
Physician	22 (8.9)
Nurse	54 (22.0)
Administrative staff	56 (22.8)
Nursing technician/health care technician	71 (28.9)
Dentist	16 (6.5)
Community health worker	27 (11.0)
Hours worked per week, n (%)	
Under 20 hours	5 (2.0)
20 to 40 hours	228 (92.7)
Above 40 hours	13 (5.3)
Years worked, n (%)	
Under one year	20 (8.1)
One to six years	88 (35.8)
Above six years	138 (56.1)

Table 2 shows the composite percent positive score of the MOSPSC and its dimensions. The overall MOSPSC composite percent positive score was 49.9 ± 14.4%. Positive patient safety culture was observed in three dimensions: Teamwork (73.1 ± 25.0%), Organizational learning (62.9 ± 33.4%), and Patient care tracking/follow-up (60.1 ± 29.8%). However, there was no dimension classified as strength. Nine dimensions were classified as weak points that require interventions to improve the patient safety culture: Overall perceptions of patient safety and quality (58.5 ± 30.9%), Overall ratings on quality and patient safety (55.4 ± 25.0%), Communication about error (53.9 ± 26.2%), Communication openness (52.5 ± 26.1%), Owner/managing partner/leadership support for patient safety (42.6 ± 32.0%), Office processes and standardization (41.8 ± 29.3), Information exchange with other institutions (39.4 ± 35.4%), Work pressure and pace (32.7 ± 18.7%), and Staff training (24.7 ± 30.7%).

**Table 2:** Composite percent positive score of the Medical Office Survey on Patient Safety Culture (MOSPSC) and its dimensions (n = 246).

MOSPSC Dimension	Mean (SD)	Median (IQ25-75%)
Overall ratings on quality and patient safety, %	55.4 (25.0)	50.0 (40.0–70.0)
Information exchange with other institutions, %	39.4 (35.4)	25.0 (0.0–75.0)
Teamwork, %	73.1 (27.4)	75.0 (50.0–100.0)
Work pressure and pace, %	32.7 (18.7)	25.0 (25.0–50.0)
Staff training, %	24.7 (30.7)	0.0 (0.0–33.3)
Office processes and standardization, %	41.8 (29.3)	50.0 (25.0–50.0)
Communication openness, %	52.5 (26.1)	50.0 (25.0–75.0)
Patient care tracking/follow-up, %	60.1 (29.8)	62.5 (50.0–75.0)
Communication about error, %	53.9 (26.2)	50.0 (25.0–75.0)
Owner/managing partner/leadership support for patient safety, %	42.6 (32.0)	50.0 (25.0–75.0)
Organizational learning, %	62.9 (33.4)	66.7 (33.3–100.0)
Overall perceptions of patient safety and quality, %	58.5 (30.9)	50.0 (25.0–75.0)
Overall, %	49.9 (14.4)	49.9 (40.7–59.6)

SD: standard deviation; 25–75% IQ: 25–75% interquartile range.

Table 3 compares the composite percent positive score of the MOSPSC and its dimensions between job roles. There was no significant difference in the composite percent positive score of overall MOSPSC between job roles (p = 0.135). However, there were significant differences between job roles and the dimensions: Overall ratings on quality and patient safety (p < 0.001), Information exchange with other institutions (p = 0.003), Teamwork (p < 0.001), Work pressure and pace (p = 0.016), Office processes and standardization (p = 0.022), Communication openness (p = 0.026), Patient care tracking/follow-up (p = 0.005), Communication about error (p = 0.012), Owner/managing partner/leadership support for patient safety (p < 0.001), and Organizational learning (p = 0.018). In the Overall ratings on quality and patient safety dimension, administrative staffs showed a significantly lower score than physicians (p < 0.001), nurses (p < 0.001), dentists (p < 0.001), nursing technicians/health care technicians (p < 0.001), and community health workers (p = 0.005), also nurses showed a significantly higher score than the nursing technicians/health care technicians (p = 0.026). In the Information exchange with other institutions dimension, only nurses compared to administrative staff showed a significant difference, with nurses showing higher scores (p = 0.001). In the Teamwork dimension, nurses presented a significantly lower score than physicians (p = 0.001), community health workers (p = 0.009), and administrative staff (p = 0.012). In the Work pressure and pace dimension, community health workers presented a significantly lower score than nurses (p = 0.009) and nursing technicians/health care technicians (p = 0.012). In the Office Processes and Standardization dimension, only physicians compared to community health workers showed a significant difference, with physicians showing lower scores (p = 0.017). In the Patient care tracking/follow-up dimension, nurses presented a significantly lower score than community health workers (p = 0.017) and administrative staff (p = 0.027). In the Communication about error dimension, only nurses compared to community health workers showed a significant difference, with nurses showing lower scores (p = 0.011). In the Owner/managing partner/leadership support for patient safety dimension, physicians showed a significantly lower score than nursing technicians/health care technicians (p < 0.001), community health workers (p < 0.001), and administrative staff (p = 0.001), also nurses showed a significantly lower score than nursing technicians/health care technicians (p = 0.019), and community health workers (p = 0.027). In the Organizational learning dimension, only nurses compared to administrative staff showed a significant difference, with nurses showing lower scores (p = 0.011). There was no significant difference in the composite percent positive score of Overall perceptions of patient safety and quality between job roles (p = 0.156).

**Table 3:** Analysis of composite percent positive score of the Medical Office Survey on Patient Safety Culture (MOSPSC) and its dimensions between job role (n = 246).

MOSPSC Dimension	Physician (n = 22)	Nurse (n = 54)	Dentist (n = 16)	Nursing technician/ Health care technician (n = 71)	Community health worker (n = 27)	Administrative staff (n = 56)	p-value
Overall ratings on quality and patient safety, %,							
mean (SD)	70.4 (20.1)	69.1 (22.8)	68.8 (27.8)	55.8 (22.3)	53.3 (20.4)	32.8 (15.8)	< 0.001
median (25–75% IQ)	70.0 (60.0–82.5)	75.0 (60.0–90.0)	75.0 (50.0–90.0)	50.0 (40.0–70)	50.0 (40.0–70.0)	30.0 (22.5–47.5)	
Information exchange with other institutions, %,							
mean (SD)	42.0 (34.8)	50.0 (31.1)	48.4 (46.1)	38.0 (38.2)	47.2 (38.8)	23.7 (25.0)	0.003
median (25–75% IQ)	25.0 (18.8–75.0)	50.0 (25.0–75.0)	37.5 (0.0–100.0)	25.0 (0.0–75.0)	50.0 (0.0–75.0)	25.0 (0.0–50.0)	
Teamwork, %,							
mean (SD)	87.5 (18.5)	59.7 (30.9)	79.7 (18.8)	69.0 (29.4)	83.3 (18.3)	78.6 (23.6)	< 0.001
median (25–75% IQ)	100.0 (75.0–100.0	50.0 (50.0–75.0)	75.0 (75.0–100.0	75.0 (50.0–100.0	75.0 (75.0–100.0)	75.0 (56.2–100.0)	
Work pressure and pace, %,							
mean (SD)	31.8 (15.8)	35.2 (15.8)	35.9 (24.1)	34.8 (18.7)	21.3 (13.3)	32.6 (21.3)	0.016
median (25–75% IQ)	25.0 (25.0–50.0)	25.0 (25.0–50.0)	25.0 (25.0–100.0)	25.0 (25.0–50.0)	25.0 (25.0–25.0)	25.0 (25.0–50.0)	
Staff training, %,							
mean (SD)	10.6 (18.9)	23.4 (26.8)	16.7 (27.2)	29.6 (32.6)	32.1 (31.3)	23.8 (32.8)	0.069
median (25–75% IQ)	0.0 (0.0–33.3)	0.0 (0.0–33.3)	0.0 (0.0–33.3)	33.3 (0.0–33.3)	33.3 (0.0–66.7)	0.0 (0.0–33.3)	
Office processes and standardization, %,							
mean (SD)	29.5 (26.3)	38.9 (27.8)	34.4 (28.7)	69.0 (29.4)	57.4 (26.7)	40.6 (31.1)	0.022
median (25–75% IQ)	25.0 (0.0–50.0)	25.0 (25.0–50.0)	37.5 (0.0–50.0)	75.0 (50.0–100.0)	50.0 (25.0–75.0)	50.0 (6.2–50.0)	
Communication openness, %,							
mean (SD)	62.5 (22.8)	50.9 (27.9)	50.0 (30.3)	45.8 (25.3)	55.6 (22.3)	58.0 (25.3)	0.026
median (25–75% IQ)	75.0 (50.0–75.0)	50.0 (25.0–75.0)	50.0 (25.0–75.0)	50.0 (25.0–75.0)	50.0 (50.0–75.0)	75.0 (50.0–75.0)	
Patient care tracking/follow-up, %,							
mean (SD)	68.2 (26.9)	48.1 (24.7)	53.1 (32.8)	60.6 (32.6)	69.4 (23.3)	65.2 (30.8)	0.005
median (25–75% IQ)	75.0 (50.0–100.0)	50.0 (25.0–75.0)	62.5 (25.0–75.0)	50.0 (50.0–100.0)	75.0 (50.0–75.0)	75.0 (50.0–100.0)	
Communication about error, %,							
mean (SD)	51.1 (31.3)	44.0 (22.7)	56.2 (26.6)	54.9 (25.9)	63.9 (26.3)	57.6 (25.6)	0.012
median (25–75% IQ)	50.0 (25.0–75.0)	50.0 (25.0–56.2)	50.0 (50.0–75.0)	50.0 (50.100.0)	75.0 (50.0–75.0)	75.0 (50.0–75.0)	
Owner/managing partner/leadership support for patient safety, %,							
mean (SD)	15.9 (16.4)	31.9 (27.2)	43.8 (39.3)	50.4 (29.7)	55.6 (33.5)	46.9 (33.1)	< 0.001
median (25–75% IQ)	25.0 (0.0–25.0)	25.0 (0.0–50.0)	37.5 (0.0–75.0)	50.0 (25.0–75.0)	75.0 (25.0–75.0)	50.0 (25.0–75.0)	
Organizational learning, %,							
mean (SD)	60.6 (33.5)	50.6 (34.1)	56.2 (31.5)	64.8 (33.3)	70.4 (26.7)	71.4 (33.9)	0.018
median (25–75% IQ)	66.7 (33.3–100.0)	50.0 (33.3–66.7)	66.7 (33.3–66.7)	66.7 (33.3–100.0)	66.7 (33.3–100.0)	66.7 (66.7–100.0)	
Overall perceptions of patient safety and quality, %,							
mean (SD)	53.4 (30.2)	39.8 (31.0)	60.9 (32.9)	53.2 (29.8)	66.7 (33.2)	63.4 (34.0)	0.245
median (25–75% IQ)	62.5 (43.8–75.0)	33.3 (16.7–66.7)	75.0 (25.0–93.8)	50.0 (25.0–75.0)	75.0 (50.0–100.0)	75.0 (31.2–100.0)	
Overall, %,							
mean (SD)	48.6 (13.8)	46.6 (10.8)	50.3 (16.6)	50.1 (14.8)	56.3 (15.2)	49.6 (15.6)	0.135
median (25–75% IQ)	50.4 (43.4–50.1)	47.3 (41.0–53.6)	49.4 (34.6–65.4)	49.3 (39.9–60.7)	53.3 (44.2–69.4)	50.5 (37.2–63.4)	

SD: standard deviation; 25–75% IQ: 25–75%: interquartile range.

**Fig 1:**
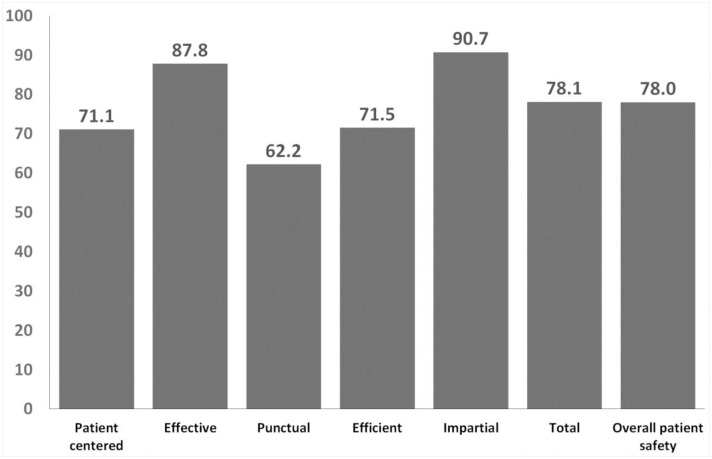
Percentage of positive responses on overall quality assessment and overall patient safety (n = 246).

Fig 1 shows the percentage of positive responses on Overall Quality Assessment (78.1%) and Overall Patient Safety Assessment (78.0%), with positive responses above 60%. Regarding the items of Overall Quality Assessment, the best assessments were observed in Impartial (90.7%) and Effective (87.8%) areas. Efficient (71.5%), Patient-Centered (71.1%), and Punctual (62.2%) areas also showed a percentage of positive responses above 60%.

Table 4 compares the percentage of positive responses between job roles/functions on Overall quality assessment and Overall patient safety assessment. Overall quality assessment showed a significant difference between job roles (p < 0.001), in which nursing technician/health care technicians showed a significantly lower score than physicians (p = 0.002), nurses (p < 0.001), dentist (p = 0.008), community health worker (p = 0.004), and administrative staff (p < 0.001). Regarding the items of the Overall quality assessment, only the Effective and Efficient areas showed a significant difference between job roles/functions and the dimensions, p < 0.001 and p = 0.001, respectively. In the Effective area, nursing technicians/health care technicians showed a significantly lower score than nurses (p < 0.001) and community health workers (p = 0.028). In the Efficient area, nursing technician/health care technicians showed a significantly lower score than physicians (p = 0.002) and nurses (p = 0.021). There was no significant difference regarding Overall patient safety assessment between job roles (p = 0.156).

**Table 4:** Comparison of the overall quality assessment and overall patient safety according to job role (n = 246).

MOSPSC Dimension	Physician (n = 22)	Nurse (n = 54)	Dentist (n = 16)	Nursing technician/ Health care technician (n = 71)	Community health worker (n = 27)	Administrative staff (n = 56)	p-value
Overall quality assessment							
Patient-centered, n (%)	17 (77.3)	37 (68.5)	12 (75.0)	43 (60.6)	25 (92.6)	41 (73.2)	0.057
Effective, n (%)	21 (95.5)	53 (98.1)	14 (87.5)	52 (73.2)	26 (96.3)	50 (89.3)	< 0.001
Punctual, n (%)	14 (63.6)	40 (74.1)	12 (75.0)	38 (53.5)	16 (59.3)	33 (58.9)	0.221
Efficient, n (%)	21 (95.5)	43 (79.6)	14 (87.5)	38 (53.5)	20 (74.1)	40 (71.4)	0.001
Impartial, n (%)	18 (81.8)	53 (98.1)	14 (87.5)	64 (90.1)	22 (81.5)	52 (92.9)	0.117
Overall, n (%)^a^	91 (82.7)	226 (83.7)	66 (82.5)	240 (67.6)	109 (80.7)	229 (81.8)	< 0.001
Overall patient safety, n (%)	16 (72.7)	43 (79.6)	12 (75.0)	60 (84.5)	16 (59.3)	56 (80.4)	0.156

^a^ Overall positive responses on the five areas (Patient-centered, Effective, Punctual, Efficient, and Impartial).

## Discussion

Our study included health care professionals from 35 primary health services in the Federal District, Brazil. The mean overall MOSPSC composite percent positive score for patient safety culture was 49.9%, which is nearly the mean observed in another study performed with PHC professionals in a municipality in South of Brazil (50.8%) and a Turkish study in PHC setting (47%) [5, 31]. On the contrary, studies conducted in Poland, Iran, Yemeni, and Greece PHC services showed a higher overall MOSPSC composite percent positive score than our study, 78.7%, 67%, 57%, and 78.2%, respectively [6, 32–34]. According to the AHRQ recommendations, only the study conducted in Yemen showed positive safety culture in the MOSPSC assessment, and none of these studies had a score above 75%. These findings suggest the need for initiatives to improve the safety culture in PHC services as already observed in other settings. A study performed in the USA demonstrated a significant increase in the patient safety culture in a six-month follow-up of programs to improve patient safety and quality. The improved patient safety culture was also associated with a significant reduction in preventable damages, serious adverse events, and adjusted hospital mortality [35].

Nine dimensions showed a negative patient safety culture assessment with a mean composite percent positive score below 60%. The Staff training and Pressure and work pace dimensions showed lower assessments when compared to other MOSPSC dimensions, with composite percent positive score means of 24.7%, 32.7%, and 39.4%, respectively. Only three dimensions, Teamwork, Organizational learning, and Patient care tracking/follow-up showed a positive patient safety culture assessment. However, they cannot be classified as a strength because the composite percent positive score means were below 75%, the AHRQ recommendation [20]. These results point out the weaknesses of the safety culture in PHC and organizational issues that should be addressed, especially promoting initiatives to strengthen the worst-scored dimensions in the MOSPSC assessment [5, 36].

Similar to our results, Teamwork and Organizational learning dimensions were also among the highest scored MOSPSC dimensions in studies performed in Turkey [31], Yemen [33], Iran [34], and Kuwait [37]. The comprehensive primary care with the organization of health care services based on teamwork and collaborative practices among the health care professionals and services is a unique feature of PHC services that promotes and strengthens teamwork [38]. Especially in the FHS, comprehensive primary care is an efficient strategy to promote collaborative work and organizational learning with positive impacts on the quality and safety of PHC services [38–40].

The staff training dimension had a lower assessment among all MOSPSC dimensions in our study (24.7%). This result was lower than observed in surveys performed in the USA [15], Poland [32], and Spain [41]. Promoting training with the joint debate is an essential strategy to improve health care quality and safety, which generally requires low-cost actions [5]. However, this result demonstrates the lack of quality improvement training to teach health care professionals about methods that could be used to analyze and improve the quality and patient safety culture in PHC services evaluated in our study [42].

Besides, the Teamwork dimension represents a strong point of initiatives to improve the culture of patient safety in PHC that is fundamental, influencing the health care professionals to remain satisfied and participatory, encouraging attitudes towards personal and professional well-being [13, 40]. Our results in this dimension were similar to the studies performed in Turkey [31], Poland [32], Iran [34], and Spain [41], and above the means of composite percent positive score of two other Brazilian studies [5, 43]. Nurses presented the lowest score in this dimension (59.7%) among job roles, significantly lower than physicians, community health workers, and administrative staff. This result contrasts with another survey about the work organizational climate with FHS professionals that showed the highest scores in nurses [40]. This aspect indicates that, although this dimension has had a positive assessment, it is vital to identify the peculiarities and needs of each job role to draw actions for further patient safety culture improvement.

The Pressure and work pace dimension is a particular issue for patient security in PHC setting since it was among the lowest score in MOSPSC dimensions in our study and also in studies performed in the USA [15], Turkey [31], Poland [32], Yemen [33], Iran [34], and Kuwait [37]. Furthermore, community health workers presented the lowest score in this dimension (21.3%) among job roles, significantly lower than nurses and nursing technicians/health care technicians. As a result, the PHC team often feels overworked and without time to promptly care for patients who need it. This issue compromises health care delivery quality and safety for patients and their families and other activities such as coordination, planning, and training [15]. Such contexts are especially for community health workers, who experience these issues as workers and community members [28]. The negative patient safety culture assessment in the Pressure and work pace dimension demonstrates the need to institute processes, technologies, and changes to improve efficiency and reduce the work pace that leads to management and team burnout [15].

The Patient care tracking/follow-up dimension showed positive safety culture. The 60.1% score in our study was better than observed in a previous Brazilian study (56.1%) [5], and studies conducted in Yemen (52%) [33], and Kuwait (41%) [37]. This dimension measures the PHC service to remind patients about appointments, documents the patients’ treatment compliance, follows up with patients who need monitoring, and follows up when reports from an outside provider are not received [15]. Once the FHS is based in family health teams assigned to cover a geographic area with each team responsible for registering every family, monitoring living conditions and health status, and providing primary care in their area, this PHC model has a more privileged position than traditional models for monitoring the patient’s health and well-being. Among job roles, community health workers are often in more privileged positions since they carry out their activities directly in the community and the patients’ homes. Each household receives at least one visit every month from dedicated community health workers, regardless of need [22, 23, 28]. Indeed, the community health workers had the highest score in the Patient care tracking/follow-up dimension, and it was significantly higher than nurses and administrative staff.

In the Overall ratings on quality and patient safety dimension, the administrative staff had a significantly lower score, significantly lower than other job roles. A USA study showed a gap between administrative support and the other team members in this respect. Integrated teams, united and willing to work together, provide safe assistance consequently reducing the chances of errors and adverse events [44]. Our result contrasts with another patient safety culture survey in a California healthcare system. They found clinicians to be more likely to provide negative responses than non-clinicians [15].

The overall patient safety assessment reached a mean of 78%, similar to another Brazilian study (79%). This result may be related to the health care professions’ satisfaction with teamwork in the PHC setting. However, compared to the negative safety culture scores in the MOSPSC, this positive assessment may also suggest that healthcare professionals misunderstand patient safety issues [45, 46]. The overall patient safety assessment drew more attention, considering that no dimension showed a score above 75%, indicating the lack of strengths regarding patient safety culture in the evaluated PHC services [20].

Regarding the overall quality assessment, all areas achieved a percentage of positive responses above 75%, except for Punctual (62.2%), which represents actions to deliver health care on time, minimizing potentially damaging delays. The Punctual lower score than other areas of quality assessment agree with the negative patient safety culture evaluation in the Pressure and work pace dimension. Impartial (90.7%) was the best-scored area of quality assessment, which means providing the same quality of care to all individuals, regardless of gender, ethnicity, socioeconomic status, language, which obtained 90%. There was no significant difference between overall quality assessments between job roles, except for nursing technicians/health care technicians who showed significantly lower scores than nurses and community health workers in the Effective area and physicians and nurses in the Efficient area. A study performed in Yemen showed lower scores in the overall quality assessment than ours, with percentages of positive responses below 50% in all aspects of quality assessment, except Impartial [33]. The Yemeni study results may be explained by the decisions in the PHC setting made chiefly only by the superior managers and the lack of patient involvement in the health care process, not being their opinions and preferences priorities, especially in the public healthcare sector [33].

Information exchange with other institutions, Office processes and standardization, Communication openness, and Communication about error dimensions also showed negative safety culture assessments. These results show the need to improve the standardization and acknowledgment of patient safety processes and strengthen communication among service members and other health system services. Studies in Turkey and Kuwait also indicate that communication is an area of concern for patient safety in PHC settings [31, 33].

Among our study limitations, the convenience sample may lead health professionals who answered the survey to be the most interested in the study topic. Second, the cross-sectional design did not establish causal relationships between the job roles and the patient safety culture scores. Other variables not evaluated in the study may have influenced these results. Third, the non-application of the MOSPSC items with open questions may have limited a better understanding of the health care professionals’ perception regarding patient safety culture. Finally, the PHC in Brasília has some singularities, compared to other Brazilian states, that may reduce the possibility of generalizing our results for all Brazilian primary health care services. However, these singularities make the PHC in Federal District an exciting setting for research. First, in 2017, a program was started to convert all PHC models adopted in the Federal District into the FHS that sought to reorganize the entire PHC structure, placing the FHS as the only model of the PHC services [29]. Second, the PHC services management is entirely provided by the Federal District Secretary of Health, including the employment of the health care professionals who joined public service through a government job competition process, which gives them more stability in the employment relationship [46]. Thus, there are no health care professionals in the Federal District PHC services employed by the Social Health Organizations, entities of the third sector that provide services under management contracts made with the direct public administration. As a result, healthcare professionals have less stable employment relationships, which is standard practice in several Brazilian municipalities [47]. In this respect, especially on sensitive questions about their perception regarding patient safety culture, professionals with the highest stable employment relationship tend to feel less pressured, and they have less biased responses than professionals with the lowest long-lasting employment relationship, such as those employed by the Social Health Organizations. Despite these limitations, our study draws attention to the weaknesses of the patient safety culture and organizational issues in PHC that should be addressed. Thus, it is recommended to encourage the exchange of information and knowledge between the team of professionals, periodic meetings to discuss the work process and clinical cases, with multidisciplinary action, permanent training with professionals working in PHC, and improvement in the management of primary care units [48]. Communication that emphasizes shared values and goals for the organization is necessary to improve the patient safety culture and quality [8].

In conclusion, the patient safety culture assessment showed a weakness in the patient safety in the PHC services. The MOSPSC and nine dimensions present a negative safety culture assessment, regardless of the high scores in the overall patient safety and quality assessments. Only Teamwork, Organizational learning, and Patient care tracking/follow-up dimensions showed a positive patient safety culture assessment. These results point out the need for initiatives to improve the safety culture in PHC services, especially in Staff training and Pressure and work pace the worst-scored dimensions.

## Supporting information

S1 File English and Portuguese MOSPSC versions. (DOCX)
