# Supplementary material for: Patient safety culture in primary health care: Medical office survey on patient safety culture in a Brazilian family health strategy setting
Source: PLoS One. 2022 Jul 26;17(7):e0271158. doi: 10.1371/journal.pone.0271158 (PMC9321378; doi:10.1371/journal.pone.0271158)
Supplement: S1 File — (DOCX) [file pone.0271158.s001.docx]

**Medical Office Survey on Patient Safety**

**SURVEY INSTRUCTIONS**

Think about the way things are done in your medical office and provide your opinions on issues that affect the overall safety and quality of the care provided to patients in your office.

►In this survey, the term **provider** refers to physicians, physician assistants, and nurse practitioners who diagnose, treat patients, and prescribe medications. The term **staff** refers to all others who work in the office.

• If a question does not apply to you or you don’t know the answer, please check “Does Not

Apply or Don’t Know.”

• If you work in more than one office or location for your practice, when answering this survey

answer only about the office location where you received this survey—do not answer about the entire practice.

• If your medical office is in a building with other medical offices, answer only about the specific

medical office where you work—do not answer about any other medical offices in the building.

**SECTION A: List of Patient Safety and Quality Issues**

The following items describe things that can happen in medical offices that affect patient safety and quality of care. **In your best estimate, how often did the following things happen in your medical office *OVER THE PAST 12 MONTHS***?

**Several** **Once or**

**times in** **twice in** **Does Not**

**Access to Care**

**Daily**

**Weekly**

**Monthly**

**the past**

**12**

**months**

**the past**

**12**

**months**

**Not in the past 12 months**

**Apply or Don’t Know**

1. A patient was unable to get an

appointment within 48 hours for an

acute/serious problem ........................ 🞎1

🞎2

🞎3

🞎4

🞎5

🞎6

🞎9

**Patient Identification**

2. The wrong chart/medical record

was used for a patient ........................ 🞎1

🞎2

🞎3

🞎4

🞎5

🞎6

🞎9

**Charts/Medical Records**

3. A patient’s chart/medical record

was not available when needed ......... 🞎1

🞎2

🞎3

🞎4

🞎5

🞎6

🞎9

4. Medical information was filed,

scanned, or entered into the wrong

patient’s chart/medical record ............ 🞎1

🞎2

🞎3

🞎4

🞎5

🞎6

🞎9

**Medical Equipment**

5. Medical equipment was not working properly or was in need of repair or replacement........................................

🞎1

🞎2

🞎3

🞎4

🞎5

🞎6

🞎9

**SECTION A: List of Patient Safety and Quality Issues (continued)**

**How often did the following things happen in your medical office *OVER THE PAST 12 MONTHS*?**

**Several** **Once or**

**times in** **twice in**

**Medication**

**Daily**

**Weekly**

**Monthly**

**the past**

**12**

**months**

**the past**

**12**

**months**

**Not in the past 12 months**

**Does Not Apply or**

**Don’t Know**

6. A pharmacy contacted our office to

clarify or correct a prescription .........

🞎1

🞎2

🞎3

🞎4

🞎5

🞎6

🞎9

7. A patient’s medication list was not

updated during his or her visit ..........

🞎1

🞎2

🞎3

🞎4

🞎5

🞎6

🞎9

**Diagnostics & Tests**

8. The results from a lab or imaging test were not available when needed...........................................

🞎1

🞎2

🞎3

🞎4

🞎5

🞎6

🞎9

9. A critical abnormal result from a lab or imaging test was not followed up within 1 business day ......................

🞎1

🞎2

🞎3

🞎4

🞎5

🞎6

🞎9

**SECTION B: Information Exchange With Other Settings**

Information Exchange With Other Settings

**Over the past 12 months, how often has your medical office had problems exchanging accurate, complete, and timely information with**:

**Problems** **Problems**

**several** **once or** **No** **Does Not**

**Problems**

**daily**

**Problems**

**weekly**

**Problems**

**monthly**

**times in**

**the past 12**

**months**

**twice in the past 12 months**

**problems in**

**the past 12**

**months**

**Apply or Don’t Know**

1. Outside labs/imaging centers? ... 🞎1 🞎2 🞎3 🞎4 🞎5 🞎6 🞎9

2. Other medical offices/ outside

physicians? ......................... .

🞎1

🞎2

🞎3

🞎4

🞎5

🞎6

🞎9

3. Pharmacies? ........................ 🞎1 🞎2 🞎3 🞎4 🞎5 🞎6 🞎9

4. Hospitals? ............................ 🞎1 🞎2 🞎3 🞎4 🞎5 🞎6 🞎9

5. Other ? (Specify): __________________________

🞎1

🞎2

🞎3

🞎4

🞎5

🞎6

🞎9

**SECTION C: Working in Your Medical Office**

**How much do you agree or disagree with the following statements?**

**Strongly**

**Disagree**

**Disagree**

**Neither**

**Agree**

**nor**

**Disagree**

**Agree**

**Strongly**

**Agree**

**Does Not Apply or**

**Don’t**

**Know**

1. When someone in this office gets really busy,

others help out............................................................

🞎1

🞎2

🞎3

🞎4

🞎5

🞎9

2. In this office, there is a good working relationship

between staff and providers .......................................

🞎1

🞎2

🞎3

🞎4

🞎5

🞎9

3. In this office, we often feel rushed when taking

care of patients ...........................................................

🞎1

🞎2

🞎3

🞎4

🞎5

🞎9

4. This office trains staff when new processes are put

into place ................................................................

🞎1

🞎2

🞎3

🞎4

🞎5

🞎9

5. In this office, we treat each other with respect ........... 🞎1 🞎2 🞎3 🞎4 🞎5 🞎9

6. We have too many patients for the number of

providers in this office ........................................

🞎1

🞎2

🞎3

🞎4

🞎5

🞎9

7. This office makes sure staff get the on-the-job

training they need .......................................................

🞎1

🞎2

🞎3

🞎4

🞎5

🞎9

8. This office is more disorganized than it should be ..... 🞎1 🞎2 🞎3 🞎4 🞎5 🞎9

9. We have good procedures for checking that work

in this office was done correctly............................

🞎1

🞎2

🞎3

🞎4

🞎5

🞎9

10. Staff in this office are asked to do tasks they

haven’t been trained to do..........................................

🞎1

🞎2

🞎3

🞎4

🞎5

🞎9

11. We have enough staff to handle our patient load....... 🞎1 🞎2 🞎3 🞎4 🞎5 🞎9

12. We have problems with workflow in this office ......... 🞎1 🞎2 🞎3 🞎4 🞎5 🞎9

13. This office emphasizes teamwork in taking care of

patients .......................................................................

🞎1

🞎2

🞎3

🞎4

🞎5

🞎9

14. This office has too many patients to be able to

handle everything effectively................................

🞎1

🞎2

🞎3

🞎4

🞎5

🞎9

15. Staff in this office follow standardized processes to

get tasks done..................................................

🞎1

🞎2

🞎3

🞎4

🞎5

🞎9

**SECTION D: Communication and Followup**

**How often do the following things happen in your medical office?**

**Never**

**Rarely**

**Some-**

**times**

**Most of**

**the time**

**Always**

**Does Not Apply or**

**Don’t**

**Know**

1. Providers in this office are open to staff ideas

about how to improve office processes ...................... 🞎1

🞎2

🞎3

🞎4

🞎5

🞎9

2. Staff are encouraged to express alternative

viewpoints in this office...............................................

🞎1

🞎2

🞎3

🞎4

🞎5

🞎9

3. This office reminds patients when they need to schedule an appointment for preventive or routine care.............................................................................

🞎1

🞎2

🞎3

🞎4

🞎5

🞎9

4. Staff are afraid to ask questions when something

does not seem right ....................................................

🞎1

🞎2

🞎3

🞎4

🞎5

🞎9

5. This office documents how well our chronic-care

patients follow their treatment plans...........................

🞎1

🞎2

🞎3

🞎4

🞎5

🞎9

6. Our office follows up when we do not receive a

report we are expecting from an outside provider......

🞎1

🞎2

🞎3

🞎4

🞎5

🞎9

7. Staff feel like their mistakes are held against them. 🞎1 🞎2 🞎3 🞎4 🞎5 🞎9

8. Providers and staff talk openly about office

problems. ..............................................................

🞎1

🞎2

🞎3

🞎4

🞎5

🞎9

9. This office follows up with patients who need

monitoring...................................................................

🞎1

🞎2

🞎3

🞎4

🞎5

🞎9

10. It is difficult to voice disagreement in this office ........ 🞎1 🞎2 🞎3 🞎4 🞎5 🞎9

11. In this office, we discuss ways to prevent errors

from happening again.................................................

🞎1

🞎2

🞎3

🞎4

🞎5

🞎9

12. Staff are willing to report mistakes they observe in

this office ....................................................................

🞎1

🞎2

🞎3

🞎4

🞎5

🞎9

**SECTION E: Owner/Managing Partner/Leadership Support**

Owner/Managing Partner/Leadership Support

**A. Are you an owner, a managing partner, or in a leadership position with responsibility for making financial**

**decisions for your medical office?**

🞎1 Yes 🡪***Go to Section F***

🞎2 No 🡪***Continue below***

**Neither**

**How much do you agree or disagree with the following statements about the owners/ managing partners/leadership of your medical office?**

**Strongly**

**Disagree**

**Disagree**

**Agree**

**nor**

**Disagree**

**Agree**

**Strongly**

**Agree**

**Does Not**

**Apply or**

**Don’t Know**

1. They aren’t investing enough resources to

improve the quality of care in this office .................... 🞎1

🞎2

🞎3

🞎4

🞎5

🞎9

2. They overlook patient care mistakes that happen

over and over............................................................. 🞎1

🞎2

🞎3

🞎4

🞎5

🞎9

3. They place a high priority on improving patient

care processes .......................................................... 🞎1

🞎2

🞎3

🞎4

🞎5

🞎9

4. They make decisions too often based on what is best for the office rather than what is best for patients ......................................................................

🞎1

🞎2

🞎3

🞎4

🞎5

🞎9

**SECTION F: Your Medical Office**

Your Medical Office

**How much do you agree or disagree with the following statements?**

**Strongly**

**Disagree**

**Disagree**

**Neither**

**Agree**

**nor**

**Disagree**

**Agree**

**Strongly**

**Agree**

**Does Not**

**Apply or**

**Don’t Know**

1. When there is a problem in our office, we see if we

need to change the way we do things ....................... 🞎1

🞎2

🞎3

🞎4

🞎5

🞎9

2. Our office processes are good at preventing

mistakes that could affect patients ............................ 🞎1

🞎2

🞎3

🞎4

🞎5

🞎9

3. Mistakes happen more than they should in this

office .......................................................................... 🞎1

🞎2

🞎3

🞎4

🞎5

🞎9

4. It is just by chance that we don’t make more

mistakes that affect our patients................................ 🞎1

🞎2

🞎3

🞎4

🞎5

🞎9

5. This office is good at changing office processes to

make sure the same problems don’t happen

again .......................................................................... 🞎1

🞎2

🞎3

🞎4

🞎5

🞎9

6. In this office, getting more work done is more

important than quality of care .................................... 🞎1

🞎2

🞎3

🞎4

🞎5

🞎9

7. After this office makes changes to improve the

patient care process, we check to see if the

changes worked ........................................................ 🞎1

🞎2

🞎3

🞎4

🞎5

🞎9

**SECTION G: Overall Rating**

***Overall Ratings on Quality***

**1. Overall, how would you rate your medical office on each of the following areas of health care quality?**

**Poor**

**Fair**

**Good**

**Very good**

**Excellent**

**a. Patient**

**centered**

Is responsive to individual patient preferences, needs, and

values .......................................

🞎1

🞎2

🞎3

🞎4

🞎5

**b. Effective** Is based on scientific knowledge 🞎1 🞎2 🞎3 🞎4 🞎5

**c. Timely**

Minimizes waits and potentially harmful delays ..........................

🞎1

🞎2

🞎3

🞎4

🞎5

**d. Efficient** Ensures cost-effective care

(avoids waste, overuse, and misuse of services) ..................

🞎1

🞎2

🞎3

🞎4

🞎5

**e. Equitable**

Provides the same quality of care to all individuals regardless of gender, race, ethnicity,

socioeconomic status, language, etc............................

🞎1

🞎2

🞎3

🞎4

🞎5

***Overall Rating on Patient Safety***

**2. Overall, how would you rate the systems and clinical processes your medical office has in place to prevent,**

**catch, and correct problems that have the potential to affect patient**

**Poor**

**Fair**

**Good**

**Very good**

**Excellent**

🞎1 🞎2 🞎3 🞎4 🞎5

**SECTION H: Background Questions**

**1. How long have you worked in this medical office location?**

🞎a. Less than 2 months 🞎d. 3 years to less than 6 years

🞎b. 2 months to less than 1 year 🞎e. 6 years to less than 11 years

🞎c. 1 year to less than 3 years 🞎f. 11 years or more

**2. Typically, how many hours per week do you work in this medical office location?**

🞎a. 1 to 4 hours per week 🞎d. 25 to 32 hours per week

🞎b. 5 to 16 hours per week 🞎e. 33 to 40 hours per week

🞎c. 17 to 24 hours per week 🞎f. 41 hours per week or more

**SECTION H: Background Questions (continued)**

**3. What is your position in this office? Check ONE category that best applies to your job.**

🞎**a. Physician (MD or DO**)

🞎**b. Physician Assistant, Nurse Practitioner, Clinical Nurse Specialist, Nurse Midwife, Advanced Practice**

**Nurse, etc.**

🞎**c. Management**

Practice Manager Business Manager

Office Manager Nurse Manager

Office Administrator

Lab Manager

Other Manager

🞎**d. Administrative or clerical staff**

Insurance Processor Front Desk

Billing Staff Receptionist

Referral Staff Scheduler (appointments, surgery, etc.)

Medical Records Other administrative or clerical staff position

🞎**e. Nurse (RN), Licensed Vocational Nurse (LVN), Licensed Practical Nurse (LPN)**

🞎**f. Other clinical staff or clinical support staff**

Medical Assistant Technician (all types)

Nursing Aide

Therapist (all types)

Other clinical staff or clinical support staff

🞎**g. Other position**; please specify: ____________________________________________________

**SECTION I: Your Comments**

**Please feel free to write any comments you may have about patient safety or quality of care in your medical office.**

***THANK YOU FOR COMPLETING THIS SURVEY.***

**Pesquisa sobre Cultura de Segurança do Paciente para**

**Atenção Primária**

**INSTRUÇÕES DA PESQUISA**

Pense sobre a maneira como as coisas são feitas no serviço de saúde onde você trabalha e dê sua opinião sobre questões que afetam a segurança e a qualidade do cuidado prestado aos pacientes.

Se uma questão não se aplica a você ou se você não sabe a resposta, por favor, marque “Não se aplica ou não sei”.

Se você trabalha em mais de um serviço de saúde, ao responder esta pesquisa, responda apenas sobre o local onde você a recebeu; não responda sobre sua prática de forma geral.

Se você trabalha em um local que possui outros serviços de saúde, responda somente em relação ao seu próprio local de trabalho.

**SEÇÃO A: Lista de questões sobre segurança do paciente e qualidade**

Os itens a seguir descrevem situações que podem ocorrer em serviços de saúde, afetando a segurança do paciente e a qualidade do cuidado. Pelos seus cálculos, com que frequência os fatos listados abaixo aconteceram em seu local de trabalho NOS ÚLTIMOS 12 MESES?

**Diariamente**

**Pelo**

**menos**

**uma vez**

**na**

**semana**

**Pelo**

**menos uma vez ao mês**

**Várias**

**vezes**

**nos**

**últimos**

**12**

**meses**

**Uma ou duas vezes**

**nos**

**últimos**

**12**

**meses**

**Não aconteceu nos últimos**

**12 meses**

**Não Se Aplica ou Não**

**Sei**

**Acesso ao cuidado**

1. Um paciente não conseguiu uma consulta em até 48 horas para um problema sério/agudo.

🞎1

🞎2

🞎3

🞎4

🞎5

🞎6

🞎9

**Identificação do Paciente**

2. ***No*** ***atendimento*** ***de*** ***um***

***paciente*** foi utilizado um

prontuário/registro de outro

paciente.

🞎1

🞎2

🞎3

🞎4

🞎5

🞎6

🞎9

**Prontuários/registros**

3. O prontuário/registro de um paciente não estava disponível quando necessário.

🞎1

🞎2

🞎3

🞎4

🞎5

🞎6

🞎9

4. Informações clínicas de um

paciente foram arquivadas,

digitalizadas ou inseridas no

prontuário/registro de outro

paciente.

🞎1

🞎2

🞎3

🞎4

🞎5

🞎6

🞎9

**Equipamento**

5. Um equipamento necessário ao

atendimento não funcionou

adequadamente ou necessitava reparo ou substituição.

🞎1

🞎2

🞎3

🞎4

🞎5

🞎6

🞎9

**SEÇÃO A: Lista de questões sobre segurança do paciente e qualidade (continuação)**

**Com que frequência os fatos listados abaixo aconteceram em seu local de trabalho NOS ÚLTIMOS 12 MESES?**

**Medicamento**

**Diariamente**

**Pelo**

**menos**

**uma vez**

**na**

**semana**

**Pelo**

**menos uma vez ao mês**

**Várias**

**vezes**

**nos**

**últimos**

**12**

**meses**

**Uma ou duas vezes**

**nos**

**últimos**

**12**

**meses**

**Não aconteceu nos últimos**

**12 meses**

**Não Se Aplica ou Não**

**Sei**

6. O paciente retornou à unidade de saúde para esclarecer ou corrigir uma prescrição.

🞎1

🞎2

🞎3

🞎4

🞎5

🞎6

🞎9

7. Os medicamentos utilizados por um paciente não foram revisados pelo profissional de saúde durante sua consulta.

🞎1

🞎2

🞎3

🞎4

🞎5

🞎6

🞎9

**Diagnósticos & Testes**

**8.** ***Os exames laboratoriais ou de***

***imagem não foram realizados quando necessário.***

🞎1

🞎2

🞎3

🞎4

🞎5

🞎6

🞎9

9. ***Os*** ***resultados*** ***de*** ***exames***

laboratoriais ou de imagem não estavam disponíveis quando

necessário.

🞎1

🞎2

🞎3

🞎4

🞎5

🞎6

🞎9

**10.** Um resultado anormal de um

exame laboratorial ou de

imagem não foi

acompanhado/avaliado em

tempo hábil.

🞎1

🞎2

🞎3

🞎4

🞎5

🞎6

🞎9

**SEÇÃO B: Troca de informações com outras instituições**

Nos últimos 12 meses, com que frequência ***este*** serviço de saúde apresentou problemas relacionados à troca de informações completas, precisas e pontuais com:

**Problemas**

**diariamente**

***Problem a pelo menos***

***uma vez***

***na***

***semana***

***Problema***

***pelo***

***menos uma vez ao mês***

**Vários**

**problema**

**s nos últimos 12**

**meses**

**Um ou dois problemas nos últimos**

**12 meses**

**Nenhum**

**problema**

**nos**

**últimos**

**12 meses**

**Não se**

**Aplica ou**

**Não Sei**

***1.*** Centros de imagem/laboratórios da rede de atenção à saúde?

🞎1

🞎2

🞎3

🞎4

🞎5

🞎6

🞎9

***2.*** Outros serviços de

saúde/médicos da rede de atenção à saúde?

🞎1

🞎2

🞎3

🞎4

🞎5

🞎6

🞎9

3. Farmácias? ............................ 🞎1 🞎2 🞎3 🞎4 🞎5 🞎6 🞎9

4. Hospitais? .............................. 🞎1 🞎2 🞎3 🞎4 🞎5 🞎6 🞎9

Outros? Por favor, especifique:

_____________________

🞎1

🞎2

🞎3

🞎4

🞎5

🞎6

🞎9

**SEÇÃO C: Trabalhando *neste* serviço de saúde**

**Quanto você concorda ou discorda com as seguintes afirmações?**

**Discordo**

**Totalmente**

**Discordo**

**Não**

**Concordo**

**Nem**

**Discordo**

**Concordo**

**Concordo**

**Totalmente**

**Não se Aplica ou Não**

**Sei**

1. Quando alguém neste serviço está muito

ocupado, outros colegas ajudam.

🞎1

🞎2

🞎3

🞎4

🞎5

🞎9

2. Neste serviço há uma boa relação de trabalho entre os médicos e demais profissionais.

🞎1

🞎2

🞎3

🞎4

🞎5

🞎9

3. Neste serviço frequentemente nos sentimos apressados ao atender o paciente.

🞎1

🞎2

🞎3

🞎4

🞎5

🞎9

4. Este serviço treina a equipe sempre que

novos processos são implantados.

🞎1

🞎2

🞎3

🞎4

🞎5

🞎9

5. Neste serviço tratamos uns aos outros

com respeito.

🞎1

🞎2

🞎3

🞎4

🞎5

🞎9

6. Neste serviço a quantidade de pacientes é muito alta em relação ao número de médicos disponíveis.

🞎1

🞎2

🞎3

🞎4

🞎5

🞎9

7. Este serviço garante que sua equipe receba atualizações necessárias ao atendimento.

🞎1

🞎2

🞎3

🞎4

🞎5

🞎9

8. Neste serviço a desorganização é maior

que o aceitável.

🞎1

🞎2

🞎3

🞎4

🞎5

🞎9

9. Neste serviço há procedimentos para verificar se o trabalho foi realizado corretamente.

🞎1

🞎2

🞎3

🞎4

🞎5

🞎9

10. Os profissionais que trabalham neste serviço são solicitados a realizar tarefas para as quais não foram treinados.

🞎1

🞎2

🞎3

🞎4

🞎5

🞎9

11. Neste serviço a quantidade de profissionais da equipe é suficiente para atender o número de pacientes.

🞎1

🞎2

🞎3

🞎4

🞎5

🞎9

12. Neste serviço há problemas com o fluxo

de trabalho.

🞎1

🞎2

🞎3

🞎4

🞎5

🞎9

13. Este serviço valoriza o trabalho em equipe

no cuidado aos pacientes.

🞎1

🞎2

🞎3

🞎4

🞎5

🞎9

14. Neste serviço há número maior de pacientes que a capacidade para atendê- los de maneira eficiente.

🞎1

🞎2

🞎3

🞎4

🞎5

🞎9

15. A equipe deste serviço segue processos padronizados para realizar suas *atividades*.

🞎1

🞎2

🞎3

🞎4

🞎5

🞎9

**SEÇÃO D: Comunicação e Acompanhamento**

Com que frequência os fatos a seguir ocorrem neste serviço?

**Nunca**

**Raramente**

**Às**

**vezes**

**Quase**

**sempre**

**Sempre**

**Não se**

**Aplica ou**

**Não Sei**

1. Os médicos deste serviço estão abertos para as ideias dos demais integrantes da equipe sobre como melhorar os processos de trabalho.

🞎1

🞎2

🞎3

🞎4

🞎5

🞎9

2. Neste serviço a equipe é incentivada a

expressar outros pontos de vista.

🞎1

🞎2

🞎3

🞎4

🞎5

🞎9

3. Neste serviço os pacientes são avisados quando precisam agendar uma consulta para cuidados preventivos ou de rotina.

🞎1

🞎2

🞎3

🞎4

🞎5

🞎

4. Neste serviço a equipe tem receio de fazer

perguntas quando algo não parece correto.

🞎1

🞎2

🞎3

🞎4

🞎5

🞎9

5. Este serviço registra a maneira como pacientes crônicos seguem o plano de tratamento.

🞎1

🞎2

🞎3

🞎4

🞎5

🞎9

6. Este serviço faz acompanhamento quando não

recebe um relatório *esperado* de outro serviço.

🞎1

🞎2

🞎3

🞎4

🞎5

🞎9

7. A equipe deste serviço acredita que seus erros

possam ser usados contra si.

🞎1

🞎2

🞎3

🞎4

🞎5

🞎9

8. A equipe fala abertamente sobre os problemas

neste serviço.

🞎1

🞎2

🞎3

🞎4

🞎5

🞎9

9. Este serviço acompanha os pacientes que

precisam de monitoramento.

🞎1

🞎2

🞎3

🞎4

🞎5

🞎9

10. Neste serviço é difícil expressar opiniões

diferentes.

🞎1

🞎2

🞎3

🞎4

🞎5

🞎9

11. Neste serviço discutimos maneiras de evitar

que erros aconteçam novamente.

🞎1

🞎2

🞎3

🞎4

🞎5

🞎9

12. Os funcionários estão dispostos a relatar erros

que observam neste serviço.

🞎1

🞎2

🞎3

🞎4

🞎5

🞎9

**SEÇÃO E: Apoio de gestores/administradores/líderes**

**A. Você é gestor/administrador ou** tem algum cargo de liderança com responsabilidade para tomar

decisões financeiras pelo serviço?

🞎1 Sim 🡪***Vá para Seção F***

🞎2 Não 🡪***Continue abaixo***

**Quanto você concorda ou discorda das seguintes afirmações sobre os gestores/líderes do seu serviço?**

**Discordo Totalmente**

**Discordo**

**Não**

**Concordo**

**nem**

**Discordo**

**Concordo**

**Concordo**

**Totalmente**

**Não se**

**Aplica ou**

**Não Sei**

1. Eles não estão investindo recursos suficientes para melhorar a qualidade do cuidado neste serviço.

🞎1

🞎2

🞎3

🞎4

🞎5

🞎9

2. Eles ignoram erros que se repetem

no cuidado aos pacientes.

🞎1

🞎2

🞎3

🞎4

🞎5

🞎9

3. Eles dão prioridade à melhoria dos processos de atendimento aos pacientes.

🞎1

🞎2

🞎3

🞎4

🞎5

🞎9

4. Eles frequentemente tomam decisões baseadas no que é melhor para o serviço e não no que é melhor para os pacientes.

🞎1

🞎2

🞎3

🞎4

🞎5

🞎9

**SEÇÃO F: Seu serviço de saúde**

**Quanto você concorda ou discorda das seguintes afirmações?**

**Discordo**

**Totalmente**

➁

**Discordo**

➁

**Não**

**Concordo**

**Nem**

**Discordo**

➁

**Concordo**

➁

**Concordo**

**Totalmente**

➁

**Não se Aplica ou Não**

**Sei**

➁

1. Quando há um problema em nosso serviço avaliamos se é necessário mudar a maneira como fazemos as coisas.

🞎1

🞎2

🞎3

🞎4

🞎5

🞎9

2. Nossos processos de trabalho são adequados para prevenir erros que poderiam afetar os pacientes.

🞎1

🞎2

🞎3

🞎4

🞎5

🞎9

3. Neste serviço acontecem erros com

mais frequência do que deveriam.

🞎1

🞎2

🞎3

🞎4

🞎5

🞎9

4. É apenas por acaso que não cometemos mais erros que afetam nossos pacientes.

🞎1

🞎2

🞎3

🞎4

🞎5

🞎9

5. Este serviço é eficiente em modificar processos de trabalho para prevenir que problemas se repitam.

🞎1

🞎2

🞎3

🞎4

🞎5

🞎9

6. Neste serviço a quantidade de atividades realizadas é mais importante que a qualidade do cuidado prestado.

🞎1

🞎2

🞎3

🞎4

🞎5

🞎9

7. Neste serviço, após realizarmos mudanças para melhorar o processo de atendimento ao paciente, avaliamos se elas funcionam.

🞎1

🞎2

🞎3

🞎4

🞎5

🞎9

**SEÇÃO G: Avaliação global**

**Avaliação global da qualidade**

**1.** No geral, como você classificaria este serviço de saúde em cada uma das seguintes áreas de qualidade de cuidados de saúde?

**Ruim**

**▼**

**Razoável**

**▼**

**Bom**

**▼**

**Muito Bom**

**▼**

**Excelente**

**▼**

**a.** Centrado no paciente:

É sensível às preferências individuais, necessidades e valores dos pacientes.

🞎1

🞎2

🞎3

🞎4

🞎5

**b.** Efetivo:

É baseado no conhecimento científico.

🞎1

🞎2

🞎3

🞎4

🞎5

**c.** Pontual:

Minimiza esperas e atrasos potencialmente prejudiciais.

🞎1

🞎2

🞎3

🞎4

🞎5

**d.** Eficiente:

Garante um cuidado de bom custo-benefício (evita o desperdício, uso excessivo e incorreto de serviços).

🞎1

🞎2

🞎3

🞎4

🞎5

**e.** Imparcial: Fornece a mesma qualidade de

cuidados a todos os indivíduos, independentemente de gênero, etnia, status socioeconômico, idioma, etc...

🞎1

🞎2

🞎3

🞎4

🞎5

***Avaliação Geral - em Segurança do Paciente***

**2.** No geral, como você classificaria os sistemas e processos clínicos que este serviço utiliza para

prevenir, identificar e corrigir problemas que tenham o potencial de afetar pacientes?

**Ruim**

**▼**

**Razoável**

**▼**

**Bom**

**▼**

**Muito Bom**

**▼**

**Excelente**

**▼**

🞎1 🞎2 🞎3 🞎4 🞎5

**SEÇÃO H: Questões sobre a prática profissional**

1. Há quanto tempo você trabalha neste serviço?

🞎a. Há menos de 2 meses 🞎d. De 3 anos a menos de 6 anos

🞎b. De 2 meses a menos de 1 ano 🞎e. De 6 anos a menos de 11 anos

🞎c. De 1 ano a menos de 3 anos 🞎f. Há 11 anos ou mais

2. Normalmente, quantas horas por semana você trabalha neste serviço?

🞎a. 1 a 4 horas por semana 🞎d. 25 a 32 horas por semana

🞎b. 5 a 16 horas por semana 🞎e. 33 a 40 horas por semana

🞎c. 17 a 24 horas por semana 🞎f. 41 horas por semana ou mais

3. Qual é o seu cargo neste serviço? Marque UMA categoria que melhor se aplica ao seu trabalho.

🞎**a.** Médico

🞎**b.** Enfermeiro

🞎**c.** Gerência

Administrador

Gerente de Enfermagem Gerente de laboratório Outro gerente ________

🞎**d.** Equipe administrativa

Registros médicos Encarregado dos agendamentos (consultas, exames, cirurgia, etc.),

Recepção Outro cargo administrativo: __________________

Recepcionista

🞎**e.** Técnico de Enfermagem

🞎**f.** Outro pessoal clínico:

Técnico de Laboratório Técnico em Saúde Bucal

🞎Odontólogo 🞎Fisioterapeuta 🞎Nutricionista

🞎Farmacêutico 🞎Psicólogo 🞎Terapeuta Ocupacional

🞎Assistente Social 🞎Agente Comunitário de Saúde

🞎Outra função. Por favor, especifique: _________________________

**SEÇÃO I – Seus comentários**

**Por favor sinta-se à vontade para fazer os comentários que você queira sobre segurança e qualidade assistencial no serviço onde você trabalha.**

***OBRIGADO POR COMPLETAR ESTA PESQUISA.***
